# Prevalence and prognostic impact of rhabdomyolysis in adults hospitalized with diabetic ketosis: a 10-year single-center cohort study

**DOI:** 10.1530/EC-25-0824

**Published:** 2026-03-03

**Authors:** Zhen Wang, Yan Zhang, Huiying Yang, Lirui Wang

**Affiliations:** ^1^ Department of Nephrology, Shanghai Baoshan District Wusong Central Hospital (Wusong Branch, Zhongshan Hospital Affiliated to Fudan University), Shanghai, China; ^2^ Department of Endocrinology, Shanghai Baoshan District Wusong Central Hospital (Wusong Branch, Zhongshan Hospital Affiliated to Fudan University), Shanghai, China; ^3^ Department of Nephrology, Tongji Hospital, Tongji University School of Medicine, Shanghai, China

**Keywords:** diabetic ketosis, rhabdomyolysis, creatine kinase, troponin I, acute kidney injury, in-hospital mortality

## Abstract

**Objective:**

To determine the prevalence and clinical impact of rhabdomyolysis (RML) among adults hospitalized with diabetic ketosis.

**Design:**

Ten-year retrospective single-center cohort study.

**Methods:**

We reviewed adults admitted with diabetic ketosis, including isolated ketosis and diabetic ketoacidosis (2015–2024). RML was defined as peak creatine kinase >1,000 U/L; patients with confirmed acute myocardial infarction were excluded. The primary endpoint was a composite poor outcome, defined as in-hospital death or discharge against medical advice (DAMA) due to critical illness with a grave prognosis. Predictors of poor outcome were assessed using Firth-corrected multivariable logistic regression.

**Results:**

Of 920 eligible patients, 18 (1.96%) developed RML. Compared with controls, patients with RML were older and more likely to have neurological comorbidities, recent falls, impaired consciousness, infection, effective serum osmolality >320 mOsm/kg, leukocytosis, higher C-reactive protein, elevated creatinine, acute kidney injury, markedly raised myoglobin, and elevated troponin I. RML was associated with a longer hospital stay, higher costs, a higher in-hospital mortality (11.1 vs 0.67%), and a higher rate of the composite poor outcome (16.7 vs 1.11%). Leukocytosis (>9.5 × 10^9^/L; adjusted OR: 4.29, 95% CI: 1.35–14.37, *P* = 0.014) and troponin I > 0.03 ng/mL (adjusted OR: 5.79, 95% CI: 1.56–21.86, *P* = 0.009) independently predicted poor outcome.

**Conclusions:**

RML is an uncommon but important complication of diabetic ketosis, conferring a higher short-term mortality, healthcare use, and composite poor outcome. Routine screening for RML and concurrent inflammatory or cardiac injury provides a crucial window for early risk stratification, enabling clinicians to optimize therapeutic strategies and resource use for high-risk individuals.

## Introduction

Diabetic ketosis is a state of metabolic decompensation that carries risks beyond acidosis, including systemic oxidative stress and multi-organ injury (1, 2). Although uncommon, rhabdomyolysis in the setting of DKA has been associated with severe systemic complications and increased mortality in hospitalized adults. While RML accompanying DKA has been sporadically reported, evidence outside DKA remains notably sparse. Most publications comprise single-case reports or small series enriched for DKA, with few cohort-level estimates for isolated ketosis, hyperosmolar hyperglycemic state (HHS), or DKA–HHS overlap (3, 4, 5). Studies in inpatient cohorts have reported RML in approximately 7% of DKA patients; however, most existing evidence has been derived from case reports or small single-center series (3, 6, 7, 8). To our knowledge, while risk factors for RML in DKA are established, large-scale studies investigating RML across the broader spectrum of ketosis presentations remain scarce. To align with clinical practice where ketosis and DKA form a continuum, we examined the entire spectrum of hyperglycemic ketosis at presentation, including isolated ketosis and DKA. Therefore, this study aimed to determine the incidence and identify predictors for RML in a large cohort of patients with diabetic ketosis, thereby providing evidence to guide early identification and intervention across this heterogeneous population.

Early identification of patients with diabetic ketosis at heightened risk of RML would enable timely implementation of proactive measures, including aggressive volume resuscitation, rigorous electrolyte correction, renal protection, and closer hemodynamic and cardiac monitoring, thereby reducing downstream complications and healthcare resource use (3, 5, 9, 10). Despite these insights, evidence regarding rhabdomyolysis specifically in diabetic ketosis remains scarce. Our group has previously investigated rhabdomyolysis in diverse clinical settings, including drug-induced case, acute pancreatitis in maintenance hemodialysis patient, and high-risk ICU cohorts, which collectively informed the design of the present study (11, 12, 13). Therefore, we conducted a 10-year retrospective cohort study to determine the prevalence and prognostic impact of rhabdomyolysis among adults hospitalized with diabetic ketosis.

Although diabetic ketoacidosis and isolated ketosis without acidosis differ in biochemical severity, they may be viewed within a spectrum of hyperglycemic decompensation. Prior studies of diabetic emergencies have demonstrated that key pathophysiological disturbances, including dehydration, hyperosmolality, electrolyte abnormalities, infection, and systemic stress, occur across different hyperglycemic states and are not confined to acidotic presentations (14). Moreover, rhabdomyolysis has been shown to be associated with hyperosmolar and metabolic stress rather than acidosis alone (4).

## Methods

The present study included patients admitted to the Department of Endocrinology between January 2015 and December 2024. The study complied with the principles of the Declaration of Helsinki and was approved by the hospital’s ethics committee (approval No. 2025-P-01). Patients and/or the public were not involved in the design, conduct, reporting, or dissemination plans of this research. We plan to disseminate findings via the hospital website and patient education activities.

For the purpose of this study, ‘diabetic ketosis’ was defined as the presence of elevated ketone bodies in blood or urine, without the metabolic acidosis requisite for a diagnosis of DKA (arterial pH < 7.3). This definition aimed to capture the broader spectrum of ketotic states in diabetes, which may not necessitate the same intensive management as DKA but still carry significant clinical risks. Patient data were extracted from medical records, including medical history, laboratory test results, hospitalization costs, clinical outcomes, and other relevant information. Missing data were imputed accordingly. The number (percentage) of imputed values and the median imputed values by group are presented in Supplementary Table S1 (see section on Supplementary materials given at the end of the article). To delineate the clinical characteristics of diabetic ketosis complicated by RML, patients with diabetic ketosis were categorized into a RML group (peak creatine kinase, CK > 1,000 U/L) and a control group (peak CK ≤ 1,000 U/L). Rhabdomyolysis was defined as a syndrome of skeletal muscle breakdown resulting in the release of intracellular contents into the circulation, operationalized in this study as a peak CK level >1,000 U/L, a threshold commonly used in clinical practice and prior epidemiological studies (15).

The primary outcome was a composite in-hospital poor outcome, defined as in-hospital mortality or discharge against medical advice (DAMA). In this study, DAMA was strictly defined as treatment discontinuation due to clinical deterioration or failure of therapy, as documented in the medical records, typically occurring when patients were critically ill and further treatment was considered unlikely to improve prognosis. Discharges for non-medical or elective reasons were excluded from outcome assessment. This composite endpoint was chosen to capture clinically meaningful adverse events and has been applied in our previous studies of rhabdomyolysis in other high-risk populations (13). Given the relatively low incidence of in-hospital mortality alone, the composite outcome also improved statistical efficiency while reflecting severe disease progression. For transparency, in-hospital mortality and DAMA were additionally analyzed and reported separately. Secondary outcomes included the incidence of acute kidney injury (AKI), length of hospital stay, and total hospitalization costs.

**Figure 1:**
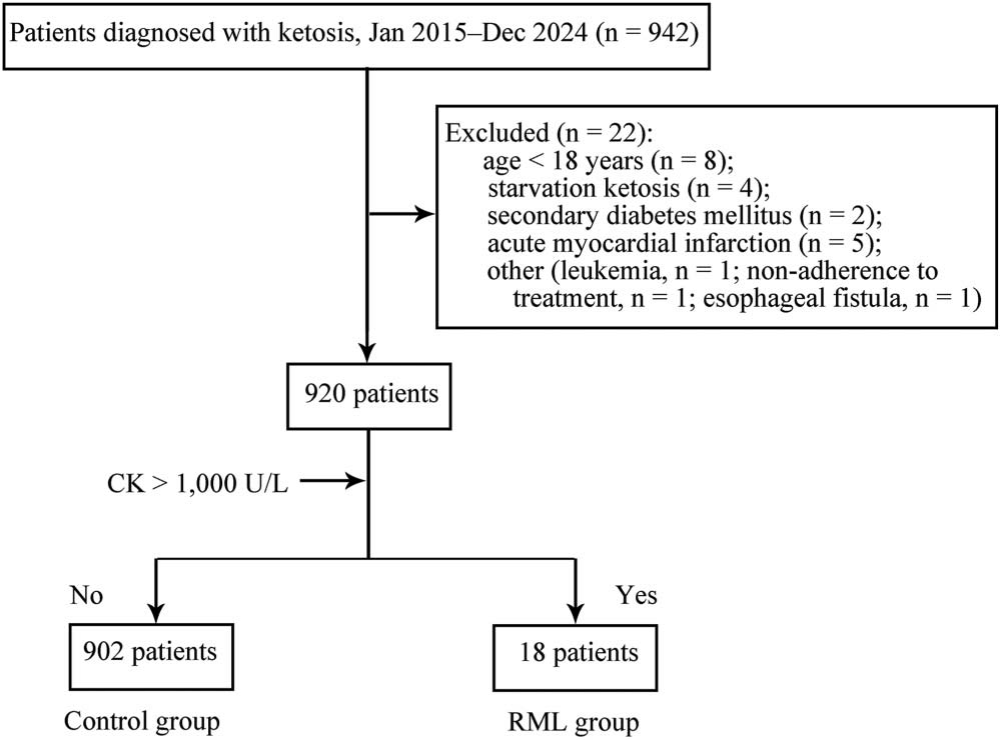
Study flow and group allocation of adults hospitalized with diabetic ketosis (January 2015–December 2024). A total of 942 patients were initially screened. Twenty-two patients were excluded based on the following criteria: age < 18 years (*n* = 8), starvation ketosis (*n* = 4), secondary diabetes mellitus (*n* = 2), acute myocardial infarction (*n* = 5), and other reasons (*n* = 3; including leukemia, non-adherence to treatment, and esophageal fistula). The remaining 920 eligible patients were stratified based on peak creatine kinase (CK) levels into two groups: the RML group (CK > 1,000 U/L, *n* = 18) and the control group (CK ≤ 1,000 U/L, *n* = 902). Abbreviations: RML, rhabdomyolysis; CK, creatine kinase.

Patients were initially identified using relevant International Classification of Diseases (ICD) diagnosis codes. To improve diagnostic accuracy, all potential cases were subsequently validated by review of laboratory data according to predefined biochemical criteria. Only patients who met both the diagnosis code-based screening and the laboratory confirmation criteria were included in the final analysis. The patient screening process, including exclusion criteria and group allocation, is illustrated in Fig. 1. Patients were excluded if they were younger than 18 years, had starvation ketosis, secondary diabetes mellitus, or acute myocardial infarction (AMI). In addition, exclusion criteria were intentionally kept narrow and mechanism-driven rather than exhaustive, in order to avoid unnecessary loss of representativeness. Conditions were excluded only when they were judged to profoundly alter metabolic status or CK levels through mechanisms unrelated to diabetic ketosis, such as leukemia, non-adherence to treatment, and esophageal fistula. To ensure the representativeness of real-world patients, other gastrointestinal conditions were not excluded unless they were deemed to directly interfere with ketone metabolism or muscle injury. Such severe gastrointestinal conditions were rare in our study cohort and thus not documented individually. Patients with AMI were excluded to avoid potential misclassification of rhabdomyolysis, as myocardial injury can independently cause substantial elevations in CK levels and is itself strongly associated with adverse in-hospital outcomes, thereby confounding both the exposure and outcome of interest (15, 16). In this study, troponin I was conceptualized as a prognostic biomarker for non-AMI myocardial injury rather than a diagnostic criterion for AMI (17).

Peak values during the index hospitalization were recorded for CK, myoglobin, and troponin I, whereas all other laboratory indicators were taken from the first measurement obtained after admission. A peak troponin I > 0.03 ng/mL is commonly used as the cutoff for defining ‘elevated’ troponin I in clinical and research settings (18). While an elevated troponin I is highly suggestive of acute coronary syndromes (ACSs), it is a non-specific finding with a wide differential diagnosis encompassing myriad cardiac and non-cardiac etiologies (19, 20). AKI was defined and staged according to the kidney disease: improving global outcomes (KDIGO) 2012 criteria, based on changes in serum creatinine from the baseline value (21). Effective serum osmolality was calculated using the following formula: 2 × (serum sodium (mEq/L) + plasma glucose (mg/dL)/18, and hyperosmolality was defined as >320 mOsm/kg H_2_O. Infection was defined as a clinical diagnosis of an infectious process (e.g., pneumonia and urinary tract infection) supported by microbiological evidence, imaging, or the initiation of antibiotic therapy by the treating physician.

Categorical variables are presented as frequencies and percentages [*n*/*N* (%)]. Continuous variables with non-normal distributions are summarized as medians with interquartile ranges (IQRs). For each variable, *N* represents the number of patients with available data. Group comparisons were conducted using the chi-square test for categorical variables (or Fisher’s exact test when expected cell counts were <5) and the Wilcoxon rank-sum test (Mann–Whitney *U*) for non-normally distributed continuous variables. A *P*-value <0.05 was considered statistically significant. To identify independent predictors of the poor outcome, we performed multivariable logistic regression using Firth’s penalized likelihood to mitigate overfitting and small-sample bias given the limited number of events (*n* = 13). A parsimonious model was specified *a priori* to preserve an acceptable events-per-variable ratio while adjusting for non-redundant pathophysiological domains, comprising the primary exposure (group: RML vs non-RML) and five clinically essential covariates: age ≥ 60 years (fundamental demographic confounder), troponin I > 0.03 ng/mL (myocardial injury), white blood cell count (WBC) > 9.5 × 10^9^/L (systemic inflammation, preferred over CRP or a clinical diagnosis of infection to avoid multicollinearity), effective serum osmolality >320 mOsm/kg (severity of hyperosmolar stress), and AKI (guideline-defined complication and key mediator, preferred over elevated creatinine as a more specific clinical endpoint). Confounding was addressed through a combination of mechanism-driven exclusion criteria, parsimonious multivariable adjustment using Firth-corrected logistic regression, avoidance of collinearity through domain-based covariate selection, and prespecified sensitivity analyses. To ensure parsimony and avoid conceptual redundancy, other variables were excluded; in particular, myoglobin was not modeled alongside group because both index skeletal muscle injury (with group defined by CK levels) and upstream vulnerability markers (e.g., fall, disorders of consciousness, and ischemic stroke) were not retained because their prognostic effects were not independent after adjustment for downstream injury and severity markers. Results are reported as aORs with 95% profile-likelihood confidence intervals and two-sided *P* values < 0.05; analyses were conducted in R (version 4.2.1) using the logistf package. Given the potential heterogeneity between acidotic and non-acidotic ketosis, we prespecified a sensitivity analysis stratified by acid–base status to assess the robustness of the primary findings.

## Results

### Cohort and group allocation

Of 942 patients initially screened between January 2015 and December 2024, 22 were excluded based on established criteria (Fig. 1). A total of 920 patients were included in the final analysis and stratified by peak CK levels. Eighteen patients (1.96%) met the definition for rhabdomyolysis (RML) (CK > 1,000 U/L), while the remaining 902 comprised the control group. Sensitivity analyses stratified by the acid–base status are shown in Supplementary Table S2. While patients with acidosis had higher rates of rhabdomyolysis and adverse outcomes, clinically relevant events were also observed among patients without acidosis, indicating that restricting analyses to DKA alone would underestimate the overall burden of rhabdomyolysis in ketotic presentations. Given the limited number of RML cases, stratified analyses were not statistically feasible.

### Baseline characteristics and clinical presentations

As shown in Table 1, patients in the RML group were significantly older than controls (mean age 69.94 ± 17.37 vs 58.09 ± 16.09 years; *P* = 0.002), and a larger proportion were aged ≥ 60 years (83.33 vs 49.11%; *P* = 0.004). The prevalence of ischemic stroke was higher in the RML group (77.78 vs 49.00%; *P* = 0.016). Falls (22.22 vs 2.32%; *P* < 0.001) and disorders of consciousness (33.33 vs 2.66%; *P* < 0.001) were also more frequent in the RML group. Diabetes duration, hypertension, coronary heart disease, and body mass index did not differ significantly between groups. Notably, and counterintuitively, self-reported weight loss was less common in the RML group than in controls (16.7 vs 48.2%; *P* = 0.008). By contrast, weight loss within the preceding 6 months did not differ significantly between the groups (16.7 vs 25.5%; *P* = 0.562).

**Table 1:** Baseline characteristics and clinical features of patients with diabetic ketosis.

	Variable	Study groups		*P* value
		Control group (*n* = 902)	RML group (*n* = 18)	
Baseline characteristics	Age, years	58.09 ± 16.09	69.94 ± 17.37	0.002
	Age ≥ 60 years	443/902 (49.11)	15/18 (83.33)	0.004
	Male sex	560/902 (62.08)	14/18 (77.78)	0.174
	Body mass index, kg/m^2^	23.67 (21.40–26.18)	23.77 (23.44–24.90)	0.222
	Diabetes duration, months	60 (2.0–156.0)	60 (0.3–195.0)	0.586
	Weight loss in past 6 months	230/902 (25.50)	3/18 (16.67)	0.562
Comorbidities	Hypertension	398/902 (44.12)	10/18 (55.56)	0.334
	Coronary heart disease	58/902 (6.43)	2/18 (11.11)	0.753
	Ischemic stroke	442/902 (49.00)	14/18 (77.78)	0.016
Clinical features and complications	Muscle pain	19/902 (2.11)	0/18 (0)	1.000
	Muscle weakness	308/902 (34.15)	9/18 (50.00)	0.161
	Dark-colored urine	0/902 (0)	0/18 (0)	1.000
	Fall	21/902 (2.33)	4/18 (22.22)	<0.001
	Disorder of consciousness	24/902 (2.66)	6/18 (33.33)	<0.001
	Acidosis	110/902 (12.20)	5/18 (27.78)	0.105
	Infection	376/902 (41.69)	14/18 (73.68)	0.002
	Acute kidney injury	108/902 (11.97)	15/18 (83.33)	<0.001
	Lipid-lowering drug use	455/902 (50.44)	7/18 (38.89)	0.339

Data are presented as mean ± SD, median (interquartile range), or *n*/*N* (%). *N* represents the number of patients with available data for each variable. RML, rhabdomyolysis; Eosm, effective osmolality; and LDL-C, low-density lipoprotein cholesterol.

Regarding complications and intercurrent conditions, infection was more common among RML patients (73.68 vs 41.69%; *P* = 0.002) and AKI occurred markedly more often (83.33 vs 11.97%; *P* < 0.001). The use of lipid-2lowering drugs at baseline was comparable between groups.

### Laboratory findings

At admission (Table 2), effective serum osmolality was higher in the RML group (median 320.08 (IQR 300.26–358.95) vs 302.96 (297.97–308.54) mmol/L; *P* < 0.001), and hyperosmolality >320 mOsm/kg was more frequent (38.89 vs 2.44%; *P* < 0.001). Markers of inflammation and leukocytosis were elevated more often in the RML group: WBC > 9.5 × 10^9^/L (50.00 vs 18.16%; *P* = 0.002) and CRP > 10 mg/L (77.78 vs 31.49%; *P* < 0.001). Elevated serum creatinine, defined as >111 μmol/L in men and >81 μmol/L in women, was also more common in the RML group (38.89 vs 15.41%; *P* = 0.018). Rates of anemia, hypoalbuminemia, transaminitis, hypercholesterolemia, and hypertriglyceridemia did not differ significantly.

**Table 2:** Laboratory findings of patients with diabetic ketosis.

	Variable	Study groups		*P* value
		Control group (*n* = 902)	RML group (*n* = 18)	
Measured at admission	HbA1c ≥ 11%	517/881 (58.68)	10/18 (55.56)	0.980
	Eosm > 320 mOsm/kg	22/902 (2.44)	7/18 (38.89)	<0.001
	Hemoglobin < 11 g/dL	107/892 (12.00)	3/18 (16.67)	0.470
	White blood cell count > 9.5 × 10^9^/L	162/892 (18.16)	9/18 (50.00)	0.002
	C-reactive protein > 10 mg/L	284/854 (33.26)	14/18 (77.78)	<0.001
	Elevated serum creatinine	139/900 (15.44)	7/18 (38.89)	0.015
	Serum albumin < 35 g/L	351/888 (39.53)	9/18 (50.00)	0.466
	ALT > 40 U/L	136/896 (15.18)	4/18 (22.22)	0.503
	Cholesterol (>5.2 mmol/L)	333/890 (37.42)	3/17 (17.65)	0.128
	LDL-C (>3.4 mmol/L)	244/889 (27.45)	2/17 (11.77)	0.179
Obtained at the time of peak CK	Peak CK, U/L	57.0 (39.0–93.25)	1,520.5 (1,153–2,784.5)	<0.001
	Myoglobin (>1,000 ng/mL)	4/902 (0.44)	8/18 (44.44)	<0.001
	Troponin I (>0.03 ng/mL)	97/894 (10.85)	12/18 (66.67)	<0.001

Data are presented as mean ± SD, median (interquartile range), or *n*/*N* (%). *N* represents the number of patients with available data for each variable. RML, rhabdomyolysis; Eosm, effective osmolality; and LDL-C, low-density lipoprotein cholesterol.

At the time of peak CK measurement, median CK was, by design, substantially higher in the RML group (1,520.5 (1,153–2,784.5) vs 57 (39–93.25) U/L; *P* < 0.001). Myoglobin >1,000 U/L (44.44 vs 0.44%; *P* < 0.001) and troponin I > 0.03 ng/mL (66.67 vs 10.75%; *P* < 0.001) were also more frequent among RML patients.

### Clinical outcomes

Compared with controls (Table 3), the RML group had a longer length of stay (13.5 (10–17) vs 10.0 (8–14) days; *P* = 0.044) and higher hospitalization costs (18.04 (15.30–22.06) vs 13.07 (10.45–16.22) × 1,000 Chinese Yuan (RMB); *P* < 0.001). All-cause in-hospital mortality was higher in the RML group (11.11% (2/18) vs 0.67% (6/902); *P* < 0.001). We observed a trend toward a higher rate of DAMA in the RML group compared with controls (5.56 vs 0.44%; *P* = 0.094), although this difference did not reach statistical significance. Accordingly, the composite endpoint of death or DAMA was more frequent in the RML group (16.67 vs 1.11%; *P* < 0.001).

**Table 3:** Clinical outcomes of patients with and without rhabdomyolysis.

	Outcomes	Study groups		*P* value
		Control group (*n* = 902)	RML group (*n* = 18)	
Primary outcome	Composite poor outcome*	10/902 (1.11)	3/18 (16.67)	<0.001
Secondary outcomes	All-cause in-hospital mortality	6/902 (0.67)	2/18 (11.11)	<0.001
	Discharge against medical advice	4/902 (0.44)	1/18 (5.56)	0.094
	Hospital stay	10 (8–14)	13.5 (10–17)	0.044
	Hospitalization costs (×1,000 RMB)	13.07 (10.45–16.22)	18.04 (15.30–22.06)	<0.001

Data are presented as *n*/*N* (%), where *N* represents the number of patients with available data for each variable, or as median (interquartile range).

* Composite poor outcome was defined as all-cause in-hospital mortality or discharge against medical advice. RML, rhabdomyolysis; RMB, Chinese Yuan.

### Predictors of poor outcome

**Figure 2:**
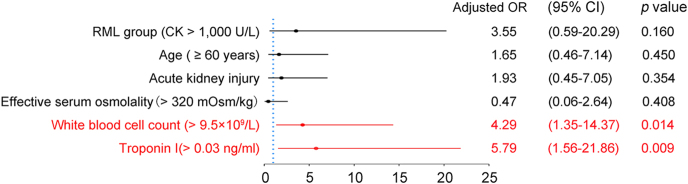
Independent predictors of poor in-hospital outcome in diabetic ketosis. Firth-corrected multivariable logistic regression in the full cohort (*n* = 920; composite outcome events = 13) evaluated associations with the composite poor outcome of in-hospital death or discharge against medical advice (DAMA). Covariates included RML group (peak CK > 1,000 U/L), age ≥ 60 years, acute kidney injury (AKI; KDIGO criteria), effective serum osmolality >320 mOsm/kg, white blood cell count (WBC) > 9.5 × 10^9^/L, and troponin I > 0.03 ng/mL. The points indicate adjusted odds ratios (aORs) with 95% confidence intervals; the red markers denote statistically significant associations (*P* < 0.05). Leukocytosis (aOR: 4.29; 95% CI: 1.35–14.37) and elevated troponin I (aOR: 5.79; 95% CI: 1.56–21.86) were significant predictors; other variables were not statistically significant. Abbreviations: RML, rhabdomyolysis; CK, creatine kinase; AKI, acute kidney injury; WBC, white blood cell count; aOR, adjusted odds ratio; CI, confidence interval; and DAMA, discharge against medical advice.

In Firth’s penalized-likelihood logistic regression analysis, several variables were assessed as potential predictors of poor outcome. Of the six variables included in the model, two emerged as statistically significant independent predictors (Fig. 2). In particular, a WBC > 9.5 × 10^9^/L was associated with a greater than fourfold increase in the odds of a poor outcome (adjusted odds ratio (aOR): 4.29, 95% confidence interval (CI): 1.35–14.37, *P* = 0.014). The strongest predictor was troponin I > 0.03 ng/mL, which was associated with an almost sixfold increase in the odds of a poor outcome (aOR: 5.79, 95% CI: 1.56–21.86, *P* = 0.009). The other variables included in the analysis did not demonstrate a statistically significant association with poor outcome. These included being in the RML group (CK > 1,000 U/L) (aOR: 3.55, 95% CI: 0.59–20.29, *P* = 0.160), age ≥ 60 years (aOR: 1.65, 95% CI: 0.46–7.14, *P* = 0.450), the presence of AKI (aOR: 1.93, 95% CI: 0.45–7.05, *P* = 0.354), and an effective serum osmolality >320 mOsm/kg (aOR: 0.47, 95% CI: 0.06–2.64, *P* = 0.408). Although not statistically significant, the point estimates for the RML group, age, and AKI suggested a trend toward an increased risk of poor outcome.

## Discussion

In this 10-year retrospective cohort of 920 adults hospitalized with diabetic ketosis, RML (defined as peak CK > 1,000 U/L) was identified in 1.96% of patients. These individuals experienced significantly worse in-hospital outcomes, including higher rates of the poor outcome (death or DAMA; 16.67 vs 1.11%) and all-cause mortality (11.11 vs 0.67%) compared to controls. Multivariable analysis confirmed that leukocytosis and elevated troponin I, but not RML itself after adjustment, were independent predictors of the poor outcome, implicating systemic inflammation and cardiac involvement as key risk determinants.

Prior studies indicate that rhabdomyolysis and related metabolic complications in diabetic emergencies are primarily associated with hyperosmolar and metabolic stress, rather than acidosis alone (4, 5, 9, 22). Accordingly, both acidotic and non-acidotic ketotic states may reasonably be examined within a shared pathophysiological framework. Observational cohort studies further show that patients commonly present along a continuum of ketotic hyperglycemia, with substantial clinical overlap between classic diabetic ketoacidosis and ketosis without overt acidosis (5, 22).

The term ‘isolated ketosis with hyperglycemia’, although not a formally standardized diagnostic label, was used as an operational definition to describe patients with clinically significant ketosis who do not meet the arterial pH criterion for diabetic ketoacidosis. This definition is consistent with prior descriptions of non-acidotic diabetic ketosis and with the continuous spectrum of ketotic decompensation recognized in clinical guidelines (9, 23). Hyperosmolar hyperglycemic state was not included, as the focus of this study was ketone-driven hyperglycemic decompensation, whereas HHS represents a mechanistically distinct condition characterized by minimal ketogenesis (4, 23).

Our findings extend prior reports that were largely limited to case series or small cohorts in DKA, in which the prevalence of RML has ranged widely and often exceeded that observed in our broader diabetic ketosis population (3, 8, 24). We also previously observed high RML rates in intensive care settings, consistent with the notion that case mix and illness severity shape RML burden (13). By quantifying RML prevalence in an unselected, single-center inpatient cohort and linking it to clinically meaningful endpoints, our study provides real-world evidence that RML, though uncommon, is a strong prognostic marker in diabetic ketosis.

The use of a composite primary outcome consisting of in-hospital mortality and DAMA merits further discussion. In the present study, DAMA was not an elective event but occurred in the setting of clinical deterioration or failure of therapy, as documented in the medical records. Clinically, both death and DAMA reflected terminal in-hospital trajectories of severe disease and an unfavorable short-term prognosis, rather than distinct or competing outcomes. Importantly, the direction and magnitude of associations were concordant when in-hospital mortality and DAMA were analyzed separately, supporting the robustness of the composite endpoint. Similar outcome definitions have been adopted in our previous studies of rhabdomyolysis in other high-risk inpatient populations (13).

Several patient-level correlates of RML emerged. Advanced age was prominent; 83.33% of RML cases were ≥60 years compared with 49.11% of controls, in line with the literature showing age-related vulnerability and higher mortality in RML (24). Biological mechanisms likely include sarcopenia, impaired mitochondrial and calcium handling in aging skeletal muscle, and prolonged immobilization after falls, which together increase susceptibility to and impede recovery from myocyte injury. The higher prevalence of prior stroke and neurological dysfunction among patients with RML suggests a contributory role for neurological impairment, as prolonged immobilization, reduced protective reflexes, involuntary muscle activity, and impaired regional perfusion may predispose skeletal muscle to ischemic injury and metabolic stress (15, 25). In addition, certain weight reduction strategies, including the use of supplements, aggressive dehydration, and perioperative factors related to bariatric surgery, may promote RML through volume depletion, electrolyte disturbances, and increased susceptibility to muscle breakdown under catabolic conditions (26). In our cohort, however, weight loss was less common in the RML group than in controls (16.67 vs 48.23%), a counterintuitive pattern that may reflect abrupt metabolic decompensation rather than chronic catabolism, or relatively preserved muscle mass providing greater substrate for injury. Similar observations have been reported in critical illness myopathy, where individuals with greater baseline muscle mass demonstrate more pronounced CK elevations during acute illness (27).

Metabolic and inflammatory derangements characteristic of diabetic crises likely contribute to RML pathogenesis. Electrolyte disturbances, especially hypophosphatemia and hypokalemia, combined with severe acidosis and hyperosmolality, set the stage for skeletal-muscle injury (28, 29). In our cohort, effective serum osmolality at admission was higher in RML patients, and a substantial proportion (38.9%) met criteria for hyperosmolality (>320 mOsm/kg). These observations align with prior reports linking hyperosmolar states to increased RML risk during diabetic emergencies (4). Beyond traumatic and metabolic etiologies, inflammatory pathways can drive severe RML. The coordinated actions of immune cells, pro-inflammatory cytokines, and complement amplify muscle-fiber injury and necrosis (30). In our cohort, RML patients more often had leukocytosis (50.00 vs 18.16%) and elevated CRP (77.78 vs 31.49%), along with a higher prevalence of infection (73.68 vs 41.69%), implicating systemic inflammation as a co-driver of muscle damage. Although the adjusted association between hyperosmolality and poor outcome was imprecise and suggested an inverse effect in our model, this estimate likely reflects small event counts, residual confounding, and collinearity rather than a true protective relationship. Clinically, effective recognition and management of hyperosmolality remain central to preventing life-threatening complications (10).

Renal and cardiac involvement was salient. AKI occurred far more often in RML than in non-RML patients (83.33 vs 11.97%), consistent with myoglobin-mediated nephrotoxicity and the established link between DKA/HHS, RML, and AKI (6, 31). Troponin I elevation was also common in RML (66.67 vs 10.75%). In the absence of obstructive coronary artery disease, troponinemia in DKA likely reflects cardiomyocyte injury from severe acidosis, lipotoxicity from elevated free fatty acids, hypovolemia, and supply–demand mismatch (20, 32). The independent association between troponin elevation and poor outcomes in our cohort underscores the need for routine cardiac monitoring and evaluation in high-risk patients, aligning with evidence that diabetic emergencies increase short-term cardiovascular events (33, 34).

Our multivariable analysis identified two independent predictors of poor outcomes: leukocytosis (aOR: 4.29) and troponin I elevation (aOR: 5.79). These markers likely represent different aspects of disease severity: systemic inflammation and cardiac involvement, respectively. The combination of these biomarkers could form the basis of a risk stratification tool to guide monitoring intensity, disposition decisions, and resource allocation. Effective management of hyperosmolality in diabetic ketosis remains crucial to prevent life-threatening complications (10). Early recognition, cautious stepwise correction, and protocol-driven fluid therapy are key to better outcomes. The lack of statistical significance after adjustment likely reflects small event numbers of model, rather than a true absence of effect; thus, it should not diminish the clinical priority of hyperosmolality management.

These observations translate into several clinical implications. First, targeted CK screening should be considered for patients with diabetic ketosis who are older or who present with neurological comorbidities, altered consciousness, falls, infection, or hyperosmolality. Second, once RML is suspected or confirmed, protocolized management is warranted: prompt isotonic fluid resuscitation, close electrolyte surveillance and replacement (with attention to phosphate and potassium), consideration of urine alkalinization in severe cases, early nephrology consultation for evolving AKI, and enhanced cardiac monitoring for patients with troponin elevation. Such measures align with contemporary guidance for hyperglycemic crises and may mitigate downstream complications and costs (5, 35).

This study has several strengths. It spans a 10-year period and includes a relatively large cohort with systematic ascertainment of clinical and laboratory variables. Both clinical and economic outcomes were evaluated. The use of a prespecified CK threshold (>1,000 U/L) ensured an objective case definition and facilitates comparison with prior studies. The comprehensive assessment of risk factors and outcomes provides actionable real-world evidence to inform clinical practice.

We acknowledge that combining DKA and non-acidotic ketosis introduces clinical heterogeneity. However, our sensitivity analyses demonstrated consistent directionality of associations across subgroups, supporting the robustness of our findings. From a pragmatic perspective, this spectrum-based approach reflects real-world inpatient practice and facilitates early identification of high-risk patients beyond classic DKA definitions. Several limitations should also be acknowledged. First, the retrospective design of this study limits causal inference and is susceptible to residual confounding and information bias, as clinical exposures, laboratory measurements, and outcomes were derived from existing medical records. Second, the relatively small number of patients with rhabdomyolysis may reduce statistical power and increase the uncertainty of effect estimates, potentially limiting the precision and robustness of our findings. In addition, the low event rate may constrain the ability to fully adjust for confounding variables and to perform more granular subgroup analyses. Finally, we excluded patients with confirmed AMI to avoid misclassification and incorporation bias (17). Collectively, these factors may limit the generalizability of our results to broader populations of adult patients with diabetic ketosis, particularly in settings with different patient characteristics or clinical practices. As a result, the applicability of these findings to broader and more heterogeneous populations of patients with diabetic ketosis should be interpreted with caution.

## Conclusions

In this 10-year, single-center cohort of 920 adults hospitalized with diabetic ketosis, RML occurred in 1.96% and was associated with a higher in-hospital mortality, a greater rate of the composite poor outcome (death or DAMA), a longer length of stay, and increased costs. In Firth-corrected multivariable logistic regression, leukocytosis (>9.5 × 10^9^/L) and troponin I > 0.03 ng/mL independently predicted poor outcome, underscoring the prognostic value of inflammatory and cardiac injury markers. Routine CK screening and early protocolized supportive care, combined with risk stratification using leukocyte count and troponin, may improve clinical decision-making and outcomes. Prospective multicenter studies are needed to validate these findings and to evaluate targeted management strategies.

## Supplementary materials



## Declaration of interest

The authors declare that there is no conflict of interest that could be perceived as prejudicing the impartiality of the work reported.

## Funding

This work was supported by grants from the following sources: the Shanghai Baoshan District Medical Key Specialty Project (Grant No. BSZK-2023-BP03) to Zhen Wang and the Baoshan District Science Popularization Project of Shanghai (Grant No. 1-L007) to Zhen Wang.

## Ethical approval and consent to participate

The study was conducted in compliance with the ethical principles delineated in the Declaration of Helsinki and was formally approved by the Ethics Committee of Shanghai Baoshan District Wusong Central Hospital (Project Number: 2025-P-01). Given the retrospective nature of this study, the Ethics Committee granted a waiver for patient consent.
