# Supplementary material for: Prevalence and prognostic impact of rhabdomyolysis in adults hospitalized with diabetic ketosis: a 10-year single-center cohort study
Source: Endocr Connect. 2026 Mar 3;15(3):e250824. doi: 10.1530/EC-25-0824 (PMC12974728; doi:10.1530/EC-25-0824)
Supplement: Supplementary file 1 [file supplementary_materials.pdf]

Table S1 The number of imputed cases and the value filled with the median of the groups

	Study Groups			
	Control group (n=902)		RML group (n=18)	
	Imputed, n (%)	Imputed value	Imputed, n (%)	Imputed value
White blood cell count	10 (1.11)	6.6 $\times 10^9$/L	0 (0)	—
Troponin I	9 (1.00)	0.01 ng/ml	0 (0)	—

The number of missing cases is reported as counts and percentages, n (%). The filled values are respectively the median of each group.

Note: "—" indicates no missing values; thus, no imputation was required.

Table S2 Comparison of rhabdomyolysis and clinical outcomes between non-acidotic ketosis and diabetic ketoacidosis

	Study Groups		
	Non-acidotic ketosis (n = 805)	Diabetic ketoacidosis (n = 115)	*p* value
Rhabdomyolysis, n (%)	13 (1.61)	5 (4.34)	0.047
All-cause in-hospital mortality	3 (0.37)	5 (4.35)	<0.001
Discharge against medical advice †	5 (0.62)	0 (0)	0.590
Composite poor outcome ‡	8 (0.99)	5 (4.35)	0.010

Categorical variables are reported as counts and percentages, n (%). Discharge against medical advice (DAMA) †: the family (or patient) elected to discontinue treatment due to the patients' critical condition and lack of improvement, resulting in discharge against medical advice. Composite poor outcome‡: includes all-cause in-hospital mortality or DAMA.
